# Driver Mutations and Single Copy Number Abnormalities Identify Binet Stage A Patients with Chronic Lymphocytic Leukemia with Aggressive Progression

**DOI:** 10.3390/jcm9113695

**Published:** 2020-11-17

**Authors:** Ana P. Gonzalez-Rodriguez, Angel R. Payer, Juan J. Menendez-Suarez, Christian Sordo-Bahamonde, Seila Lorenzo-Herrero, Joud Zanabili, Ariana Fonseca, Ana Julia Gonzalez-Huerta, Pilar Palomo, Segundo Gonzalez

**Affiliations:** 1 Department of Hematology, Hospital Universitario Central de Asturias (HUCA), 33011 Oviedo, Spain; apayer.angel@gmail.com (A.R.P.); joudzas88@gmail.com (J.Z.); FONSECAMOURELLE@hotmail.es (A.F.); anajuliagh@gmail.com (A.J.G.-H.); pilarpalomo@gmail.com (P.P.); 2 Instituto Universitario de Oncología del Principado de Asturias (IUOPA), 33006 Oviedo, Spain; Christiansbl87@gmail.com (C.S.-B.); seilalorenzoherrero@gmail.com (S.L.-H.); segundog@uniovi.es (S.G.); 3 Instituto de Investigación Sanitaria del Principado de Asturias (ISPA), 33011 Oviedo, Spain; 4 Departamento de Biología Funcional, Inmunología, Universidad de Oviedo, 33006 Oviedo, Spain; menendez.juanjo@gmail.com

**Keywords:** chronic lymphocytic leukemia, Binet, next-generation sequencing, whole exome sequencing, genetics, mutation, single copy number abnormality

## Abstract

The correlation between progression and the genetic characteristics of Binet stage A patients with chronic lymphocytic leukemia (CLL) detected by whole exome sequencing (WES) was analyzed in 55 patients. The median follow-up for the patients was 102 months. During the follow-up, 24 patients (43%) progressed. Univariate Cox analysis showed that the presence of driver mutations, the accumulation of two or more mutations, the presence of adverse mutations, immunoglobulin heavy chain genes (*IGHV*) mutation status and unfavorable single copy number abnormalities (SCNAs) were associated with a higher risk of progression. Particularly, the occurrence of an adverse mutation and unfavorable SCNAs increased the risk of progression nine-fold and five-fold, respectively. Nevertheless, only the occurrence of adverse mutations retained statistical significance in the multivariate analysis. All patients carrying an unfavorable mutation progressed with a median progression-free survival (PFS) of 29 months. The accumulation of two or more mutations also increased the risk of progression with a median PFS of 29 months. The median PFS of patients with unfavorable SCNAs was 38 months. Combining mutations and SCNAs, patients may be stratified into three groups with different prognostic outcomes: adverse (17% probability of five-year PFS), protective (86% probability of five-year PFS) and neither (62% probability of five-year PFS, *p* < 0.001). Overall, the analysis of the mutational status of patients with CLL at an early stage of the disease may allow the identification of patients with a high risk of progression. The feasibility of an early therapeutic intervention in these particular patients requires further investigation.

## 1. Introduction

Rai and Binet established a clinical staging system based on physical examination and blood count cells that has widely been used for clinical management of chronic lymphocytic leukemia (CLL) for decades [1]. However, most patients are currently diagnosed at an early stage, and a significant portion of these patients will experience an aggressive disease [2,3,4]. To refine the risk stratification, several prognostic factors have been established in recent decades, including cluster of differentiation 38 (CD38), zeta-chain-associated protein kinase 70 (ZAP-70), immunoglobulin heavy chain genes (*IGHV*) mutation status and some chromosomal abnormalities [5,6,7,8,9]. Particularly, deletion 13q14 (del(13q)) has been correlated with a more indolent disease, whereas deletion 11q22.3 (del(11q)) and deletion 17p13 (del(17p)) have been associated with a more aggressive disease and resistance to treatment [9,10]. Recently, an international prognostic score for asymptomatic early-stage CLL has been established [11]. Along the same lines, next-generation sequencing (NGS) techniques have led to the discovery of several unidentified genes that are mutated in CLL, providing invaluable information about the mechanisms involved in the pathogenesis of this disease and therapy resistance. Most CLL patients carry mutations in one or more genes, particularly patients with aggressive disease [12,13]. Multiple population-based studies and clinical trials have showed the adverse prognostic value of some mutated genes including *TP53*, *ATM*, *SF3B1*, *NOTCH1*, *BIRC3* and *ZNF292* [4,14,15,16,17,18,19,20,21,22]. Clonal acquisition of these gene mutations is an important mechanism of disease progression and drug resistance [23]. Thus, NGS is becoming a new sensitive tool in clinical practice.

Herein, we analyzed the correlation between the mutational status and single copy number abnormalities (SCNAs) detected by whole exome sequencing (WES) in a group of 55 consecutive, asymptomatic and untreated Binet stage A patients with CLL, who have been followed up for nearly 10 years.

## 2. Patients and Methods

Fifty-five consecutive, untreated, Binet stage A patients with CLL recruited at Hospital Universitario Central de Asturias between February 2011 and August 2012 were analyzed (Table 1). Clinical data were updated in April 2020. Clinical characteristics of patients (such as age, sex, Rai and Binet stages, treatment or death) and blood cell counts were obtained. Laboratory parameters and expression of CD38 and ZAP-70 on CLL cells were determined by flow cytometry [24]. Interphase fluorescence-in situ hybridization (FISH) analysis for del(17p), del(11q), trisomy 12 (tri(12)) and del(13q) was performed with peripheral blood lymphocytes. These patients were a cohort of patients sequenced by Puente et al. [25]. Briefly, leukemic cells were purified from fresh or cryopreserved mononuclear cells using magnetically labeled antibodies (AutoMACS; Miltenyi Biotec, Bergisch Gladbach, Germany). Purity was assessed by flow cytometry. WES was performed as previously reported [26]. Mutations and somatic copy number alterations (SCNAs) were analyzed using the Sidron pipeline [25]. Data acquisition and analysis were performed in compliance with protocols approved by the Ethic Committee of Principado de Asturias (ethical approval number 53/16). Written informed consent was obtained from all participants prior to study.

## 3. Statistics

Progression-free survival (PFS) and overall survival (OS) were defined as the intervals between WES study and clinical progression (defined according to International Workshop on CLL (iwCLL) recommendations), or death. Survival was analyzed using the approach of Kaplan and Meier, and the curves of each group were compared with a long rank test. Univariate and multivariate Cox proportional hazards models were used to examine the relationship between patient characteristics and survival. Comparison between survival probabilities was evaluated by Wilcoxon test. Receiver-operating characteristic analysis (ROC-curve) was plotted, and the area under the curve was calculated. The *p*-values < 0.05 were considered significant. Statistical analysis was performed using IBM SPSS statistics version 25.0 (2017).

## 4. Results

Fifty-five consecutive, untreated, Binet stage A patients with CLL were analyzed in this study (Table 1, Table S1). WES detected 42 patients to have SCNAs. The most frequent of them was del(13q), which was found in 35 patients (63%), but only 31 of them (56%) carried del(13q) as the sole aberration, and only those ones were considered to carry a “protective SCNA” [9,27,28]. Five patients (9%) had an “adverse SCNA” (tri(12) in 3 patients, del(17p) in 2 patients and del(11q) in 1 patient). Driver mutations were identified in 24 patients (43%). *PAX5* enhancer mutations were detected in 7 patients (13%); *SF3B1* mutations in 6 patients (10%); *TP53* and *CHD2* mutations in 3 patients (5%); *BIRC3*, *NOTCH1*, *CNOT3*, *ZNF292*, *KLHL6* and *SETD2* mutations in 2 patients (3%); and *ARID1A*, *HIST1H1B*, *ATM, POT1*, *BCOR*, *XPO1*, *FBXW7*, *MGA* and *CCND2* mutations were found in 1 patient (1%). Six out of twenty-four mutated patients (25%) accumulated 2 mutations; 2 patients had 3 mutations, and 1 patient carried 7 mutations. Eleven patients (20%) carried at least one adverse mutation (in *NOTCH1*, *TP53*, *SF3B1* or *BIRC3* genes), and 5 patients (9%) had protective mutations (in *CHD2* or *KLHL6* genes). The combination of SCNAs and mutations allows for further stratification of patients into three groups: adverse (those who carried adverse mutations or SCNAs) (~22%), favorable (those who carried protective mutations or SCNAs and lack adverse ones) (56%) and patients lacking all of them (~22%) (Table 1). Of note, 5 out of 7 unmutated *IGHV* patients were in the adverse group.

The median follow-up of patients was 102 months (92–110 months). During the follow-up, 24 patients (43%) progressed, and 18 patients (32%) died. PFS and OS were not reached, but five-year PFS was 65% and OS was 82% (standard error 0.01). The correlation between genetic characteristics and PFS was analyzed by the Cox regression model. In univariate analysis, the occurrence of driver mutations, *IGHV* mutation status, the accumulation of two or more mutations, the presence of adverse mutations and unfavorable SCNAs were associated with a higher risk of progression (Table 2). Particularly, the occurrence of an adverse mutation and an unfavorable SCNA increased the risk of progression nine-fold and five-fold, respectively. Nevertheless, only the occurrence of adverse mutations retained statistical significance in the multivariate analysis.

**Figure 1:**
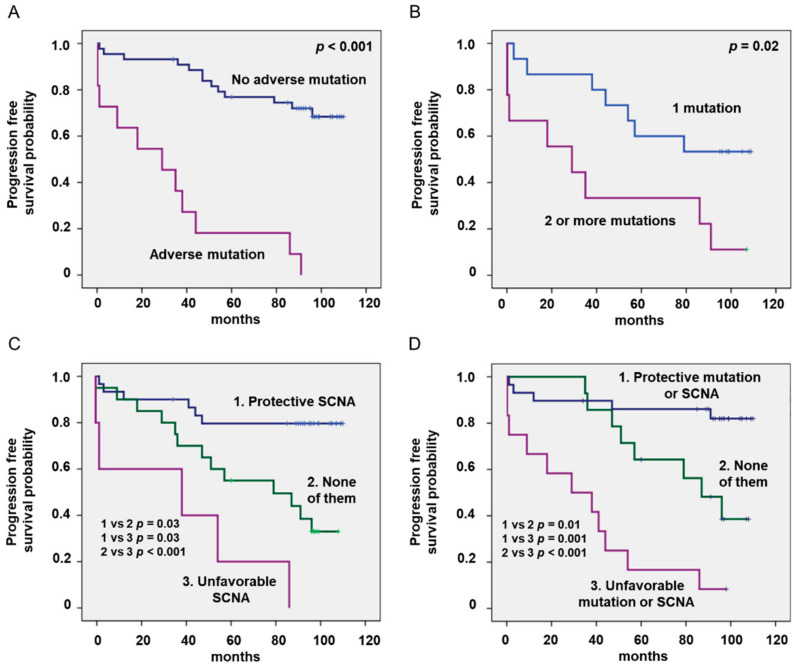
Kaplan–Meier curves of patients with chronic lymphocytic leukemia (CLL) stratified by the presence of adverse mutations (**A**), the accumulation of 2 or more mutation (**B**), single copy number abnormalities (SCNAs) (**C**) and combined prognostic groups (**D**).

Kaplan–Meier analyses showed that mutations and SCNAs had a strong impact on progression (Table 3, Figure 1). Fifteen out of twenty-four patients (62%) carrying driver mutations progressed (median PFS of 57 months, 50% probability of five-year PFS), compared with only nine patients (29%) without mutations (median PFS not reached, 77% probability of five-year PFS) (*p* = 0.001). Of note, all patients carrying an unfavorable mutation (*n* = 11) progressed (median PFS of 29 months, 18% probability of five-year PFS), but only 13 out of 44 patients (29%) without adverse mutations progressed (PFS not reached; 77% probability of five-year PFS, *p* < 0.001). The accumulation of mutations also significantly impacts survival (median PFS not reached in patients with one mutation vs. 29 months in those with two or more mutations; 71% vs. 33% probability of five-year PFS, *p* = 0.001). PFS of patients with unfavorable SCNAs was 38 months (20% probability of five-year PFS), but it was not reached in those with favorable SCNAs (80% probability of five-year PFS) and 97 months in those patients lacking both of them (55% probability of five-year PFS) (Table 3, Figure 1D). Combining mutations and SCNAs, patients may be stratified into three prognostic groups: adverse (17% probability of five-year PFS), protective (86% probability of five-year PFS) and neither (62% probability of five-year PFS). The ROC-curve of this combining model was 0.83 (CI 95%: 0.71–0.94), and it was larger than that of the driver mutations (0.73; CI 95%: 0.59–0.87) or SCNAs (0.78; CI 95%: 0.65–0.91), showing a higher predictive value.

## 5. Discussion

CLL is a highly heterogeneous malignancy with some patients showing an indolent disease for years, and others displaying an aggressive disease with a rapid progression that requires early therapeutic intervention. Staging systems, such as the Binet and Rai systems, remain useful in defining disease extent and prognosis [1]; however, at diagnosis most patients are currently at Binet stage A, and this staging system has become insufficient to finely predict the outcome of an individual patient [2,3,4]. Recently, NGS techniques have led to the precise definition of the genomic landscape in CLL, and several somatic mutations that significantly impact progression and survival of patients with CLL have been identified [13,23,25]. Similarly, NGS provides higher resolution than FISH; nevertheless, NGS and FISH give complementary information, because the clone size defines different outcomes [29,30]. A simple question raised by these recent genetic advances is how these mutations should be incorporated into the current management of patients.

Here, we performed the first study analyzing the effect of driver mutations and SCNAs detected by WES on the progression of asymptomatic and untreated consecutive patients with CLL at Binet stage A diagnosed in a single hospital. Remarkably, a long follow-up of more than 100 months was carried out. As expected, patients showed a good outcome, with a median PFS and OS that was not reached during the follow-up. However, among them there were patients with quite a diverse evolution, ranging from a rapid and aggressive progression to a long asymptomatic disease. This diversity correlates with a great variety of genetic and cytogenetic alterations. Around 40% of patients carried at least one driver mutation, with 45% of these mutated patients having an adverse mutation and 41% of them more than one mutation. Ten percent of patients carried an adverse SCNA. Univariate analysis showed that both mutations and SCNA alterations deeply influence the progression of the disease. Particularly, adverse mutations increased the risk of progression mutations nine-fold, and this was the only variable that retained statistical significance by multivariate analysis. Further, patients that carried an adverse mutation or accumulated more than one mutation displayed an aggressive disease with a median PFS of 29 months. Due to the lower frequency of recurrent mutations in patients with CLL, patients were grouped by the presence of a protective mutation (in *CHD2* and *KLHL6* genes) or favorable SCNA (del(13q) as the sole SCNA), and those with an adverse mutation (in *NOTCH1*, *TP53*, *SF3B1* or *BIRC3* genes) or unfavorable SCNA (del(17p) or del(11q)). Combining mutations and SCNAs, patients may be stratified into three prognostic groups with quite different prognostic outcomes. The 17% probability of five-year PFS observed in patients with an adverse mutation or SCNA sharply contrasts with the 86% observed in patients with protective ones.

Only patients with active disease, symptomatic disease or advanced Rai and Binet stage are currently treated. However, our study showed that asymptomatic patients at Binet stage A with adverse genetic features rapidly progress. Clinical trials may be specifically designed to analyze whether this specific group of patients may benefit from an early or specific therapeutic intervention [31,32]. Overall, routine analysis of the mutational status of patients with CLL at an early stage of disease may allow the identification of those patients with a high risk of progression who will need a strict follow-up [20]. The feasibility of an early therapeutic intervention in these particular patients requires further investigation.

## Figures and Tables

**Table 1:** Clinical and biological characteristics of patients with chronic lymphocytic leukemia (CLL).

Parameter	
Age (median)	70.5 years
Sex m/f	27 (49.1%)/28 (50.9%)
Rai stage 0/I	38 (69.1%)/17 (30.9%)
ECOG 0/1	53 (96.4%)/2 (3.6%)
*IGHV* status	Mutated: 46 (83.6%) Unmutated: 7 (12.7%) Indeterminate: 2 (3.6%)
CD38 pos/neg	8 (14.5%)/47 (85.5%)
ZAP-70 pos/neg	4 (7.4%)/50 (92.6%)
FISH caryotype	No alteration: 14 (25.5%) Del(13q): 33 (60%) Del(17p): 4 (7.3%) Trisomy 12: 2 (3.6%) Del(11q): 1 (1.8%) Complex Karyotype: 0 (0%)
SCNA (detected by WES)	No alteration: 13 (23.6%) Del(13q): 35 (63.3%) Trisomy 12: 3 (5.4%) Del(17p): 2 (3.6%) Del(11q): 1 (1.8%) Others (G2p16 (1); L18p (1); L6q15 (2); G2p26 (1); G5q34 (1); G8q24 (1); L3p21.31 (1); G3q26 (1); L15p26 (1); L20p (1)
Driver mutations (pos/neg)	24 (43.8%)/31 (56.4%)
Type of mutation	Adverse mutation: 11 (20%) Protective mutation: 5 (9.1%) Other mutations: 8 (14.5%)
Number of mutations	0: 31 (56.4%) 1: 15 (27.3%) 2: 6 (10.9%) 3: 2 (3.6%) 7: 1 (1.8%)
Genetic and SCNA groups	Adverse: 12 (21.8%) Protective: 31 (56.3%) Neither: 12 (21.8%)

m: Male; f: Female; pos: Positive; neg: Negative; SCNAs: Somatic copy number alterations; WES: Whole exome sequencing; *IGHV*, immunoglobulin heavy chain genes; FISH, fluorescence-in situ hybridization; ECOG, Eastern Cooperative Oncology Group; CD38, cluster of differentiation 38; ZAP-70, zeta-chain-associated protein kinase 70. Unfavorable mutations: *NOTCH1, TP53, ATM, SF3B1, BIRC3* and *ZNF292*. Favorable mutations: *CHD2* and *KHKL6*.

**Table 2:** Univariate analysis for progression-free survival.

Variable	Hazard Ratio	CI 95%	*p*
Driver mutations	2.73	1.19–6.26	0.017
*IGHV* mutation status	3.78	1.39–10.32	0.0009
2 or more mutations	4.81	2.03–11.38	0.001
SCNAs (unfavorable)	5.08	1.84–14	0.002
Mutations (adverse)	9.17	3.9–21.59	<0.0001

SCNAs: Somatic copy number alterations; CI 95%: Confidence interval 95%.

**Table 3:** Five-year progression-free survival (PFS) and five-year overall survival (OS) of patients stratified by the presence of genetic and cytogenetic alterations.

	Number of Patients	Five-Year PFS Probability *	*p*	Five-Year OS Probability *	*p*
Driver Mutations	Yes = 24	0.5 (0.1)	0.001	0.79 (0.08)	ns
	No = 31	0.77 (0.08)		0.87 (0.06)	
Unfavorable Mutations	Yes = 11	0.18 (0.12)	<0.001	0.73 (0.13)	0.01
	No = 44	0.77 (0.06)		0.86 (0.05)	
Number of Mutations	One = 15	0.71 (0.07)	0.001	0.85 (0.05)	0.03
	2 or more = 9	0.33 (0.16)		0.78 (0.14)	
SCNA	Unfavorable = 5	0.2 (0.18)	0.002	0.4 (0.22)	<0.001
	None = 20	0.55 (0.11)		0.8 (0.09)	
	Favorable = 30	0.8 (0.07)		0.93 (0.05)	
Prognostic Groups	Unfavorable = 12	0.17 (0.11)	0.03	0.67 (0.14)	0.02
	None = 12	0.62 (0.13)		0.77 (0.11)	
	Favorable = 31	0.86 (0.007)		0.93 (0.05)	

* Percentage (standard deviation); ns: Non-significant.

## Supplementary Materials

The following are available online at https://www.mdpi.com/2077-0383/9/11/3695/s1, Table S1: Characteristics of patients with chronic lymphocytic leukemia (CLL).
