# Supplementary material for: Driver Mutations and Single Copy Number Abnormalities Identify Binet Stage A Patients with Chronic Lymphocytic Leukemia with Aggressive Progression
Source: J Clin Med. 2020 Nov 17;9(11):3695. doi: 10.3390/jcm9113695 (PMC7698623; doi:10.3390/jcm9113695)
Supplement: Supplementary file 1 [file jcm-09-03695-s001.pdf]

Table S1. Characteristics of patients with chronic lymphocytic leukemia (CLL).

Patient	Driver mutations	IGHV status	SCNA	Progression (months)	Survival (months)
1		mutated	13q14het	no	alive
2		mutated		51	51
3	*BCOR*; *NOTCH1* 3´UTR	unmutated	13q14hom; G2p16; L18p	18	alive
4		mutated	13q14hom	no	alive
5		mutated		47	46
6		mutated	13q14het	no	alive
7	*PAX5* enhancer	mutated		79	79
8	*PAX5* enhancer	indeterminate	13q14het	no	alive
9	*KLHL6*	mutated	tri12	54	54
10		mutated	13q14het	no	alive
11		mutated	13q14het	no	alive
12		mutated		87	87
13		mutated	13q14het	no	alive
14	*POT1*; *PAX5* enhancer	mutated	13q14het	no	alive
15		mutated	13q14hom	41	99
16	*SF3B1*; *TP53* del; *ZNF292* del	unmutated	17p;L6q15;G2p16;G3q26;G5q34;G8q24	1	82
17		mutated	13q14het	no	alive
18		indeterminate	13q14het	12	12
19	*NOTCH1*; *SF3B1*	unmutated		0	alive
20		mutated	13q14het	47	47
21		mutado		no	alive
22	*PAX5* enhancer	mutated	13q14het	no	alive
23		mutated	13q14hom	no	alive
24	*SF3B1*; *TP53* del; *XPO1*; *ARID1A*; *FBXW7*; *MGA*; *SETD2*	unmutated	17p	0	36
25		unmutated	13q14het	no	alive
26	*CNOT3*	unmutated		no	alive
27	*PAX5* enhancer	mutated	13q14het	no	alive
28		mutated	13q14het; L6q15	no	alive
29	*ATM*	mutated	13q14het	44	alive
30	*BIRC3* del; *PAX5* enhancer and promoter	mutated	11q; tri12	86	86

31	*KLHL6*	mutated	13q14hom	No	alive
32		mutated	13q14het	No	alive
33		mutated	13q14het	No	alive
34		mutated		36	alive
35		mutated	13q14het	1	alive
36	*ZNF292*; *SETD2* del; *SF3B1*	unmutated	13q14het; L3p21.31	29	55
37	*SF3B1*	mutated	tri12; tri18	38	38
38		mutated	13q14het	No	alive
39	*TP53* del; *CHD2*	mutated	13q14hom; G2p16; L18p	91	91
40	*PAX5* enhancer	mutated		No	alive
41	*HIST1H1B*	mutated		57	57
42	*BIRC3*; *CNOT3*	mutated		35	68
43		mutated	13q14het	no	alive
44		mutated	13q14het	no	alive
45	*CCND2*	mutated	13q14het	3	69
46		mutated	13q14hom	no	alive
47		mutated	13q14het	no	alive
48		mutated		96	96
49	*CHD2* del	mutated	G3q26; L15q26	no	alive
50		mutated	13q14het	no	alive
51		mutated	13q14het	no	alive
52		mutated		no	alive
53	*CHD2*	mutated	13q14het	no	alive
54	*SF3B1*	mutated	L20p	9	alive
55		mutated	13q14het	no	alive

SCNA: somatic copy number alteration; *IGHV*, immunoglobulin heavy chain genes.
